# The Effect of Leaders’ Coaching Behaviors on Employee Learning Orientation: A Regulatory Focus Perspective

**DOI:** 10.3389/fpsyg.2020.543282

**Published:** 2020-10-28

**Authors:** Wei Liu, Shuting Xiang

**Affiliations:** ^1^Business School, Central University of Finance and Economics, Beijing, China; ^2^School of International Business, Southwestern University of Finance and Economics, Chengdu, China

**Keywords:** leaders’ coaching behaviors, encourage-to-explore behaviors, guide-to-learn behaviors, employee learning orientation, regulatory focus

## Abstract

Although scholars have recognized leaders’ importance in facilitating employee learning, the potential role that leaders’ specific behaviors play in followers’ learning outcomes remains elusive. Based on the literature, we identified two styles of leaders’ coaching behaviors that may promote followers’ learning, namely encourage-to-explore behaviors and guide-to-learn behaviors. We expect that these two styles of coaching behaviors would have different relationships with employee learning orientation, moderated by employees’ regulatory focus. To test our hypotheses, we conducted two studies: an experimental study using scenarios of leader behavior with data collected from 124 undergraduate students (study 1), and a field study with data from 334 pairs of employees and their direct supervisors (study 2). Results showed that encourage-to-explore behaviors had a positive relationship with employee learning orientation, whereas guide-to-learn behaviors had an inverted U-shaped relationship with employee learning orientation. When employees had low levels of promotion regulatory focus, encourage-to-explore behaviors showed a positive correlation with employee learning orientation. However, when they had low levels of prevention regulatory focus, employee learning orientation increased as the guide-to-learn behaviors increased; this increased up to an intermediate level and then decreased. As such, this study provides novel theoretical and empirical insights into leadership and learning literature.

## Introduction

Employees’ continuous development at work is an essential prerequisite for individual success and organizational effectiveness, especially in competitive and dynamic business markets (Maurer et al., 2002; Bezuijen et al., 2010). Employees with learning orientation are more likely to engage in developmental activities and challenging tasks (Redmond et al., 1993), so considerable researchers have focused on employee learning orientation and conceptualized this as the dedication and willingness to develop individual competence (Gong et al., 2009). Research suggests that learning orientation is beneficial for individuals in acquiring knowledge and skills, promoting higher performance, and enhancing creativity (Brett and VandeWalle, 1999; Kozlowski et al., 2001; Gong et al., 2009). Concerning the benefits, scholars are making efforts to explore factors that facilitate employee learning orientation, including leadership (Chi and Huang, 2014; Shariq et al., 2018), high-commitment human resource management (Yang et al., 2016; Runhaar et al., 2019), and personal traits (Debicki et al., 2016).

A growing number of studies on learning orientation insist that employee learning orientation can be promoted or precluded by managerial and contextual factors (Bezuijen et al., 2009, 2010). As managerial coaches and facilitators in the daily processes of work, leaders on the frontline should take this increasingly critical responsibility willingly and effectively (Ellinger et al., 2011). Researchers have already begun to appreciate the importance of leaders in facilitating employee learning orientation (Bezuijen et al., 2009; Shojaei et al., 2016). As a popular management tool, workplace coaching has been demonstrated to be effective for employees. Meta-analysis studies have proved that coaching has an overall positive impact on outcome criteria, including employee performance, well-being, work attitudes, and goal-directed self-regulation (Theeboom et al., 2014), and affective, skill-based, and individual-level results outcomes (Jones et al., 2016). Some empirical studies have also indicated the key role of coaching in facilitating employees’ learning (Agarwal et al., 2009; Matsuo, 2018). However, prior studies mainly treat coaching as a set of behaviors and fail to distinguish functions according to different behaviors (Kim et al., 2013; Steelman and Wolfeld, 2018). Therefore, research is needed to investigate the effect of different coaching behaviors. Accordingly, in this study, we aim to advance a novel theoretical perspective by differentiating two models of coaching behaviors for promoting employee learning orientation, namely encourage-to-explore and guide-to-learn behaviors, and examine whether certain behaviors are functional or dysfunctional for employees. Encourage-to-explore behaviors indicate that leaders expect and encourage followers to engage in exploration or discovery learning, where minimal guidance is provided. Guide-to-learn behaviors refer to leaders providing employees with specific direction, learning frames and feedback on tasks. Further, we intend to investigate how these two different models of leader coaching behaviors influence employee learning orientation separately.

To better understand the influence of leader coaching behaviors on employee learning orientation, it is helpful to delve into the relevant boundary conditions. As learning motivation and outcomes vary for different individuals, no particular learning environment or promotion behavior of leaders can be suitable for all employees (Liem et al., 2008). Employees’ learning activities are based on their beliefs (Lin et al., 2017), especially their learning motivation (Parsloe and Leedham, 2009). Therefore, we believe that regulatory focus, which distinguishes two types of individual motivational orientation, namely promotion focus and prevention focus (Higgins, 1997, 1998), is the feasible factor to examine the effectiveness of leader coaching behaviors. To explore the boundary effect of employees’ regulatory focus, we draw on the *complementary fit perspective*, which indicates that the weakness or need of an employee can be compensated by strength of external factors (Kristof, 1996). The leaders’ coaching behavior can create a context that compensates what is lacking or needed for employees with a specific regulatory orientation (Kark and Van-Dijk, 2007). The creation of the context is a product of the leader’s communications with employees, including framing the standards of goals, descriptions of appropriate behavior, and provision of feedback (Sue-Chan et al., 2012).

Our research produces three key contributions to the literature. First, by identifying two specific coaching behaviors of leaders, we further integrate and extend the manager-as-coach literature. Previous studies tend to focus on a single leadership style without distinguishing functions in coaching activities (Steelman and Wolfeld, 2018), but our research differentiates two models of coaching behaviors, namely encourage-to-explore and guide-to-learn behaviors. Second, we contributed to active learning literature by emphasizing the role of leaders in learning interventions. Scholars have commonly investigated the effect of learning interventions in a broad environment (i.e., Keith and Frese, 2005; Bell and Kozlowski, 2008), while neglect how leaders play the role in these interventions. Our research thus fills in this gap. Third, by looking at the interacting effects of leaders’ coaching behaviors and employees’ regulatory focus on employees’ learning orientations, we seek to expand the scope of existing theory and clarify the conditions under which coaching could influence employee learning orientation more effectively by applying complementary fit theory.

## Literature Background and Hypotheses

### Leaders’ Coaching Behaviors

Since the concept of coaching was introduced in the field of management by Mace (1950), the classifications and operationalizations have been discussed extensively in the existing literature (Sue-Chan et al., 2012; Dahling et al., 2016). Considerable studies regarding leaders’ behaviors show that coaching serves as a situational prime that can induce recipients to pursue the same goal in different ways (Higgins, 1997, 1998; Sue-Chan et al., 2012). Coaching is typically referred to a set of behaviors. For example, Redshaw (2000) defined coaching as giving guidance, encouragement, and support. Heslin et al. (2006) suggest that effective coaching behaviors include guidance, facilitation, and inspiration. Throughout coaching literature, two components of coaching have been commonly emphasized, including exploration encouragement and guidance.

Exploration and guidance have also been emphasized in the literature on active learning. To facilitate employees’ willingness to acquire knowledge and develop competence at work, an active learning environment includes components of exploration, guiding, and emotional control. Among all those components, exploration and guiding have been recognized as important parts of the direct leader’s responsibilities (Bezuijen et al., 2009). The need that “foster learning at and through work has far-reaching consequences for managers, who are expected to manage the workplace as a place fit for learning” has been gradually realized (Coetzer, 2007, p. 417). Leaders’ coaching can be regarded as the main source of learning context for employees (Sue-Chan et al., 2012), and the management and policies must work considering leader’s behavioral function (Ellinger et al., 2011). Accordingly, leaders may help develop their employees by encouraging and guiding. These two models of leader behaviors also represent the most frequently adopted methods in the managerial and coaching process (Redshaw, 2000; Heslin et al., 2006).

To integrate research regarding coaching behaviors and active learning, two specific coaching behaviors have been identified, namely encourage-to-explore and guide-to-learn behaviors. The encourage-to-explore behaviors imply that the leaders expect and encourage followers to engage in exploration or discovery learning, where minimal guidance is provided (Naveh et al., 2015). As the main dimensions of transformational leadership, intellectual stimulation and inspirational motivation emphasize the importance of energizing followers through novel and compelling approaches and encouraging followers to broaden their visions (Gong et al., 2009). From this perspective, some studies have mostly focused on how broadly explorative composites of leadership influence learning outcomes. For example, Shojaei et al. (2016) argued that transformational leadership which brings a change in attitude, beliefs, and values of followers will lead to achievement beyond expectations, and actions intended to improve an individual’s competence. Bezuijen et al. (2009) found that expectations from leaders were related to employee’s engagement in learning activities.

The guide-to-learn behaviors are characterized by leaders’ specified directions for employees’ learning strategy, where employees engage in systematic and pre-planned learning activities. In this model, leaders provide directions, goals, and learning frames to employees, as well as information and feedback on employees’ learning progress, which help employees to understand what else they should learn (Button et al., 1996; Dragoni, 2005; Bell and Kozlowski, 2010). However, previous research on the association between guiding behaviors and employee learning did not provide consistent conclusions. Based on the Pygmalion model, Bezuijen et al. (2009) explored the effect of leaders on employees learning through goal setting, and they found that leaders’ feedback was negatively related to employee engagement in learning activities. However, Pan et al. (2011) found that leader mentoring was positively related to personal learning and employee self-efficacy had a moderating effect on the relationship.

### Effects of Leaders’ Coaching Behaviors on Employee Learning Orientation

Based on Atkinson’s (1964) achievement motivation theory, scholars have identified goal orientation as a motivational orientation that influences individuals’ interpretations and behaviors in achievement situations (Dweck and Leggett, 1988). The goal orientation reflects individuals’ self-development beliefs and how those beliefs influence interpretation and engagement with their environment. Some scholars have classified goal orientation into two categories: learning goal orientation and performance goal orientation (Dweck, 1986; Heyman and Dweck, 1992). Learning orientation has been defined as an internal mindset that motivates individuals to be concerned with and dedicated to developing their competence (Dweck, 1986, 2000; Dweck and Leggett, 1988). Individuals with a learning orientation would seek challenges that pursue more learning opportunities (Ames and Archer, 1988).

We expect that by encouraging employees to explore, leaders may enhance employees’ intrinsic motivation to learn, and thus, promoting their learning orientation. Specifically, when employees are encouraged to engage in active exploration by leaders, they can initiate learning activities and control learning processes without any specific instructions, and determine when and how to acquire new knowledge and expand their skills all by themselves (Taris et al., 2003; Bell and Kozlowski, 2010). In this way, they will be more likely to attribute their learning activities to internal factors rather than external determinants (Deci and Ryan, 1980). Previous research has also shown that intrinsic motivation can be enhanced through enactive exploration (Wood et al., 2000; Debowski et al., 2001). With a higher level of intrinsic motivation to learn, employees’ learning orientation will be enhanced (Leung et al., 2012).

Additionally, leaders’ encourage-to-explore behaviors may deliver learning information and cues to legitimize learning behaviors for employees, thereby enhancing their learning orientation. Specifically, employees commonly use social cues to explain the behaviors of the people around them and adjust their behaviors according to the information they obtained (Salancik and Pfeffer, 1978). When leaders encourage employees to engage in explorative activities such as independently seeking new ways to improve professionally, employees may acquire the information that self-oriented exploration is needed in their work. Therefore, they would engage in more exploration to develop their competences, and this tendency may enhance their learning orientation. Accordingly, we hypothesize as follows:

Hypothesis 1: Leaders’ encourage-to-explore behaviors are positively related to employee learning orientation.

However, the relationship between guide-to-learn behaviors and employee learning orientation does not seem to be linear, and the guidance from leaders would promote employee learning orientation only to a certain extent. When leaders show low to moderate extent of guide-to-learn behaviors, employees will be provided with specific direction, goals, and learning frames, which may raise their aspiration and confidence to seek more learning opportunities (Debowski et al., 2001; Bell and Kozlowski, 2008). Additionally, they may also receive instruction and feedback on their performance from leaders, which could help them minimize errors and avoid failure during learning processes (Dweck, 1986). This may help them to learn and develop their competences more effectively (Naveh et al., 2015). Therefore, when they receive guidance from leaders, employees will be more likely to learn due to their enhanced learning confidence and competence, which may breed intrinsic motivation to learn and further enhance their learning orientation.

Nevertheless, when leaders provide too many instructions in learning activities and detailed feedback to employees, employee learning orientation may be subsequently reduced due to their decreased intrinsic motivation to learn. Specifically, when leaders raise too specific requirements, expectations, and instructions, employees may need to learn in the systematic and pre-planned manner directed by their leaders. In this way, they may lose autonomy in learning processes and tend to attribute their learning to complying with external leaders’ requirements rather than intrinsic motivation (Debowski et al., 2001). With the rise in external motivation and decline in intrinsic motivation, employees may exhibit lower learning orientation (Leung et al., 2012).

In addition, high amounts of feedback may also impair employee learning orientation due to the shift in attention focus. Specifically, when leaders provide too much feedback to employees, feedback interventions would move employees’ attention closer to the self-goal of reducing feedback-standard discrepancy and further away from the task (Kluger and Denisi, 1996). Accordingly, when received too much feedback from leaders, employees may struggle to narrow down the feedback-standard discrepancy rather than paying attention to the learning activity. This shift of attention focus may aggravate their external attribution to learn and further impair learning orientation. Accordingly, we hypothesize as follows:

Hypothesis 2: The relationship between leaders’ guide-to-learn behaviors and employee learning orientation is curvilinear (inverted U-shaped).

### The Moderating Effects of Employee Regulatory Focus

#### Complementary Fit Between Leaders’ Coaching Behaviors and Employees’ Regulatory Focus

How and when leaders affect and interact with recipients is not well understood because employees’ personal attributes also play a vital role in this relationship. Due to personal characteristics, different individuals would show different learning intentions even when they encounter similar leader behaviors. Several studies have suggested that learning environments interact with personal attributes to influence learning outcomes (Bell and Kozlowski, 2008; Gully and Chen, 2010). For example, Naveh et al. (2015) found that individual traits of openness to experience and conscientiousness play significant moderating roles in the relationship between active learning environment and errors in employees’ effort.

Within the research on exploring personal attributes, the individual regulatory focus is a critical variable that explains the motivational process of employee learning orientation. The hedonic principle, that is, people approach pleasure and avoid pain, has been the basic motivational principle in psychological theories. According to Higgins (1997, 1998), regulatory focus has been defined as the process in which people seek to align themselves with appropriate goals or standards (Brockner and Higgins, 2001). Higgins (1997) argued that people pursue two different kinds of regulatory foci in the process with which they approach pleasure and avoid pain: promotion focus and prevention focus. Individuals, who are promotion focused, are motivated by growth and development needs. They try to align themselves with their ideal selves, including their hopes, wishes, and aspirations. Individuals, who are prevention focused, are prompted by security needs, and they attempt to align themselves with their ought selves, including their duties, obligations, and responsibilities (Brockner and Higgins, 2001; Kark and Van-Dijk, 2007).

**FIGURE 1:**
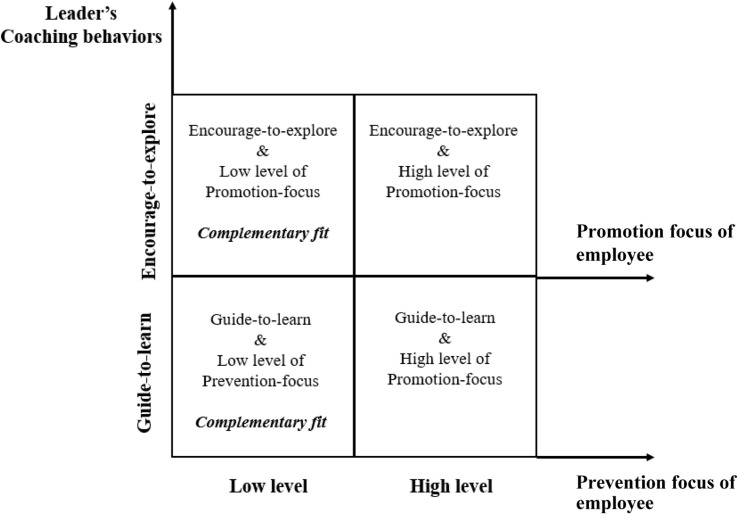
Complementary fit between leaders’ coaching behaviors and employee regulatory focus.

We propose that an individual’s regulatory focus will moderate the relationship between leaders’ coaching behavior and employee learning orientation from the perspective of complementary fit. As indicated by Kristof (1996), “fit is defined as the compatibility between people and organizations that occurs when: (a) at least one entity provides what the other need, or (b) they share similar fundamental characteristics, or (c) both” (pp. 4–5). When two entities possess different characteristics and at least one entity provides what the other entity needs or wants, complementary fit occurs (Kristof, 1996; Greguras and Diefendorff, 2009). The complementary fit between employees’ “needs” and environmental “supplies” will lead to positive outcomes (Harrison, 1978). Accordingly, we believe that leaders’ coaching behaviors will compensate employees with low levels of regulatory focus (see in Figure 1) and further affect their learning orientation. Detailed explanations are provided below.

#### Complementary Fit Between Encourage-to-Explore Behaviors and Promotion Focus

Promotion focused people are more likely to notice and recall information relating to success (Higgins and Tykocinski, 1992) by showing high motivation and persistence in tasks (Shah et al., 1998), pursuing a more explorative processing style, considering alternative hypotheses (Friedman and Förster, 2001; Righetti et al., 2011), and using these approaches as a development strategy (Kark and Van-Dijk, 2007).

Employees with high level of promotion focus will proactively show willingness to take risks (Kark and Van-Dijk, 2007) and exhibit “exploratory” behaviors such as creativity and innovation (Förster et al., 2004). They will commonly exhibit greater intrinsic motivation for engaging in learning activities. However, when encouraged to explore by leaders frequently, employees may start to attribute their learning behaviors to the external factors, namely their leader’s encourage-to-explore behaviors. With the rise of external motivation and the decline of intrinsic motivation, their learning orientation will decrease (Bakker et al., 2012). As such, the influence of encourage-to-explore behaviors on learning orientation will be limited for the employees who have high level of promotion focus.

By contrast, due to their low needs for growth and development, employees with low level of promotion focus will be less inclined to learn spontaneously. They commonly show less intrinsic motivation and persistence towards tasks and engage less in explorative activities (Higgins, 1997; Shah et al., 1998; Sue-Chan et al., 2012). Therefore, complementary fit occurs when leaders’ encourage-to-explore behaviors compensate what they have lacked in learning motivation. Specifically, leaders deliver the information and expectation to expand knowledge and skills through encourage-to-explore behaviors, which help employees increase confidence and engagement in the learning process (Noe et al., 2010). Furthermore, when expected and encouraged to explore by leaders, low prevention focused employees can initiate their learning activities and determine the learning processes (Bell and Kozlowski, 2010). Instead of receiving specific instructions from their leaders, they initiate their learning activities all by themselves. In such a way, they may start to learn and attribute their learning behaviors to internal motivation. Therefore, leaders’ encourage-to-explore behaviors compensate for their less internal motivation to learn, thus promote their learning orientation. Accordingly, we hypothesize as follows:

Hypothesis 3: Employee promotion focus moderates the relationship between leaders’ encourage-to-explore behaviors and employee learning orientation, such that the effect will be more positive for employees with low level of promotion focus.

#### Complementary Fit Between Guide-to-Learn Behaviors and Prevention Focus

People who are prevention focused tend to pay attention to information that will help them avoid failure and negative outcomes (Lockwood et al., 2002); require concrete and specific task-relevant information (Friedman and Förster, 2001); and are conservative regarding risky activities, including exploration and innovation (Förster et al., 2004).

For employees with low level of prevention focus, leaders’ directions, instructions, and detailed feedback may complement their security needs and compensate their lack of disposition to learn. In this regard, the relationship between leaders’ guide-to-learn behaviors and employees’ learning orientation would be positive. However, this positive relationship may only exist up to a certain extent because employee may feel less intrinsic motivation and more pressure to learn when leaders exhibit too many guide-to-learn behaviors (Debowski et al., 2001). Thus, it leads to a negative relationship between leaders’ guide-to-learn behaviors and employees’ learning orientation.

Provided with guide-to-learn coaching, employees with high prevention focus may also perceive complementary fit similar to those who have low levels of prevention focus. However, strain and stress caused by leaders’ guide-to-learn behaviors may exhibit strong negative effect than the benefits which fit brings. Specifically, employees with high level of prevention focus commonly show stronger intention to avoid negative outcomes and risky activities (e.g., learning). When leaders provide specific instructions and feedback toward learning activities, those employees may feel strain and stress because they are forced to do what they normally avoid (Edwards, 1996). Moreover, since people with high prevention focused are more conservative about learning, they may follow a leader’s instructions and suggestions to learn much more carefully instead of actively seeking learning opportunities. Therefore, they are more likely to attribute learning activities to external motivations, instead of intrinsic motivation (Gagné and Deci, 2005). Less intrinsic motivation to learn would lead to less subsequent learning orientation (Leung et al., 2012). To summarize, the more guide-to-learn behaviors their leaders show, the less learning orientation focus will be obtained for employees with high prevention focus. Accordingly, we hypothesize as follows:

Hypothesis 4: Employee prevention focus moderates the relationship between leaders’ guide-to-learn behaviors and employee learning orientation such that when the level of prevention focus is low, there is an inverted U-shaped relationship between guide-to-learn behaviors and employee learning orientation. When the level of prevention focus is high, there is a negative association between guide-to-learn behaviors and employee learning orientation.

**FIGURE 2:**
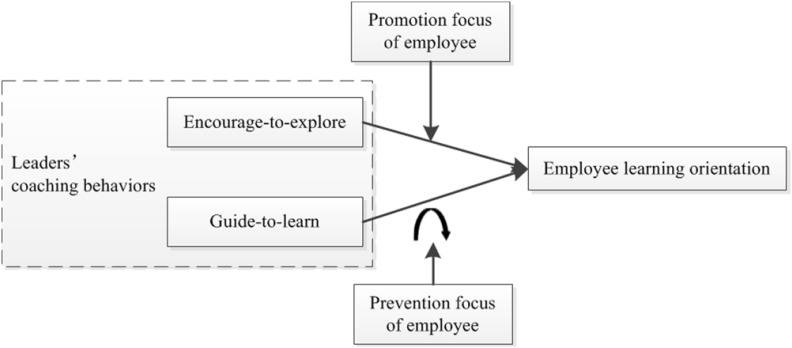
Research model.

The overall framework encompassing these four hypotheses is demonstrated in Figure 2.

## Materials and Methods

We conducted two studies to test our hypotheses. In Study 1, we test the hypotheses using scenarios of leader behavior with the data collected from undergraduate students. In Study 2, we test the hypotheses with paired data from employees and their direct leaders working in a state-owned enterprise.

### Study 1

#### Sample and Procedure

We employed two 2 × 2 between-subjects experiments (experiment 1a and 1b) online. Experiment 1a aimed to test the interactive effect between encourage-to-explore behavior (more or less) and promotion focus (low or high). Experiment 1b was designed to test the interactive effect between guide-to-learn behavior (more or less) and prevention focus (low or high). We recruited 61 participants in experiment 1a and 63 participants in experiment 1b. Participants were undergraduate students and postgraduate students majoring in management from a university in China.

Although it is ideal to manipulate regulatory focus and previous studies have shown effective ways to manipulate promotion focus versus prevention focus (e.g., Pham and Avnet, 2004; Kirmani and Zhu, 2007), it is not common to manipulate regulatory focus at different levels (e.g., high/low level of promotion focus, high/low level of prevention focus). Therefore, at the beginning of the experiment, we asked participants in each experiment to rate themselves on the promotion/prevention scale. Then, we categorized them into groups with different levels of regulatory focus by comparing their ratings with the median. For example, in Experiment 1a, those who obtained lower promotion focus ratings than the median of all participants’ ratings were labeled as having “low level of promotion focus.” Accordingly, in each experiment, half of the participants were labeled as having “low level of promotion focus (*n* = 30)/prevention focus (32)” and half were labeled as having “high level of promotion focus (*n* = 31)/prevention focus (31)”.

After a quick categorization by using our designed program, participants in groups with high/low level of regulatory focus were randomly and equally distributed in two scenarios. They were asked to complete questionnaires following a vivid image for themselves in this specific context of the scenario. In Experiment 1a, participants’ average age was 21.31 (ranging from 18 to 26). Among them, 39.3% were male and 60.7% of them were female. In experiment 1b, the average age of the participants was 20.683 (ranging from 18 to 23), where 42.9% were male and 57.1% were female.

#### Scenarios and Manipulations

##### Leaders’ Coaching Behaviors

According to the use of scenarios in leadership studies (e.g., Christie et al., 2011; Kark et al., 2015), two scenarios were developed based on items of measurement in study 1. The scenarios described a similar work situation but had different leader’ coaching behaviors: one scenario used encourage-to-explore behaviors and the second used guide-to-learn behaviors. The scenarios were as following:

##### Encourage-to-Explore Behaviors

Suppose you are working at company A, which specializes in producing high-tech products. Patent-inventing is the major responsibility for employees. Since you have entered the company, Li Lei, the leader of your R&D team, is your direct supervisor. In your work, Li Lei, instead of providing you with specific work guidance and working plan, (frequently/occasionally) encourages you to explore how to complete your assignments more effectively and efficiently. On the one hand, for the tasks you are already familiar with, he (frequently/occasionally) motivates you to reflect on novel working methods to enhance your professional competence. On the other hand, he (frequently/occasionally) inspires you to make efforts to cope with emerging problems and tasks, during which mistakes or failures will be considered as necessary steps toward acquiring new knowledge. In addition, Li Lei always (frequently/occasionally) encourages you to keep learning advanced knowledge and skills and to try putting them into practice during work.

##### Guide-to-Learn Behaviors

Suppose you are working at company A, which specializes in producing high-tech products. Patent-inventing is the major responsibility for employees. Since you have entered the company, Li Lei, the leader of your R&D team, is your direct supervisor. In your group meeting, Li Lei (frequently/occasionally) clearly informs every group member of the work assignments, project requirements, and existing problems of the group. He also (frequently/occasionally) makes detailed working plans for everyone and attempts to ensure everyone is on board through repeated communication. During work, Li Lei (frequently/occasionally) keeps a close eye on his employees and offers specific guidance on how to complete the assignments more effectively and efficiently based on his experiences. Meanwhile, Li Lei (frequently/occasionally) records your work performance and progress, and then, he provides timely feedback regarding current shortcomings and how to make improvements.

(For those who were assigned to the scenario of more coaching behaviors, we chose the word “frequently” in parentheses; “Occasionally” in parentheses was applied for participants who were assigned to the scenario of less coaching behaviors).

##### Manipulation Check

To assess the scenarios’ content validity, at the end of the scenario, participants were asked to indicate their perception on leaders’ coaching behavior on a seven-point Likert-type scale, ranging from not at all (1) to completely (7). The questionnaires were adapted from Naveh et al. (2015) study. The encourage-to-explore behaviors were assessed on six items, including “The leader encourages the followers to try new things at work” and “The leader frequently encourages the followers to seek by themselves new ways to improve professionally.” The guide-to-learn behaviors were assessed on five items, including “The leader frequently instructs the followers on how to improve their performance” and “The leader frequently gives the followers feedback on what else they need to learn.” The α-coefficient for encourage-to-explore behaviors was 0.888 and for guide-to-learn behaviors was 0.841.

Results of a *t*-test analysis showed that the ratings of encourage-to-explore behavior are higher in high EE scenario group (*N* = 31, *M* = 6.306, SD = 0.574) than in low EE scenario group (*N* = 30, M = 4.828, SD = 1.006), *t* (59) = 7.375, *p* < 0.001. The scores for guide-to-lead behavior are higher in high GL scenario group (*N* = 32, *M* = 6.281, SD = 0.422) than in low GL scenario group (*N* = 31, *M* = 4.968, SD = 0.517), *t* (61) = 11.037, *p* < 0.001. These indicated good manipulations.

#### Measures

Since all original scales are in English, we applied translation and back-translation to confirm the accuracy of the translation into Chinese (Brislin, 1986).

##### Regulatory Focus of Employees

Promotion/Prevention scales were adapted from Lockwood et al. (2002). Promotion focus was assessed with nine items, including “I frequently imagine how I will achieve my hopes and aspirations” and “I often think about the person I would ideally like to be in the future.” Prevention focus was assessed with nine items, including “In general, I am focused on preventing negative events in my life” and “I am anxious that I will fall short of my responsibilities and obligations.” The respondents responded to the items using a seven-point Likert-type scale (1 = “strongly disagree”; 7 = “strongly agree”). The α-coefficient for employee promotion focus was 0.859 (in Experiment 1a) and the α-coefficient for employee prevention focus (in Experiment 1b) was 0.758.

##### Employee Learning Orientation

We adopted Gong et al. (2009) six-item scale to assess individual learning orientation. Sample items included “I prefer tasks that really challenge me so I can learn new things” and “I desire to completely master my job.” The respondents responded to the items using a seven-point Likert-type scale (1 = “strongly disagree”; 7 = “strongly agree”). The α-coefficient for employee learning orientation was 0.730 (in experiment 1a) and 0.674 (in experiment 1b).

##### Control Variables

The control variables used in study 1: (a) gender, (b) age (in years), and (c) trait anxiety, which controlled the level of emotional control in Bell and Kozlowski’s (2008) model. Naveh et al. (2015) insisted that the level of each individual’s anxiety should be considered when investigating the potential association between level of anxiety and learning, as the learning process may be difficult and stressful.

#### Results

The means, standard deviations, and correlations among the variables in study 1 are presented in Table 1. To confirm the random assignment of individual regulatory focus to the scenario conditions, we conducted a MANOVA with the scenario conditions as independent variables. Results demonstrated a non-significant effect in both experiment 1a [*F*(1,59) = 1.642, *ns*] and experiment 1b [*F*(1,61) = 0.525, *ns*], showing that there were no significant differences for individual regulatory focus between each scenario conditions in two experiments.

**TABLE 1:** Means, standard deviations, and correlations (Study 1).

Experiment 1a	Encourage-to-Explore Behaviors								
	LEE M (SD)	MEE M (SD)	*F*(1,59)	1	2	3	4	5	6
1. EE	/	/	/						
2. Promotion focus	5.033 (0.980)	5.301 (0.617)	1.642	0.165					
3. Learning orientation	5.228 (0.724)	5.560 (0.425)	4.792*	0.274*	0.528***				
4. Gender	0.267 (0.450)	0.516 (0.508)	4.113*	0.2554*	–0.053	–0.047			
5. Age	21.833 (1.859)	21.000 (0.775)	5.286*	−0.287*	–0.117	–0.219	–0.227		
6. Trait anxiety	4.067 (1.461)	4.161 (1.440)	0.137	0.033	–0.001	–0.183	–0.088	–0.039	

**Experiment 1b**	**Guide-to-Learn Behaviors**								
	**LGL M (SD)**	**MGL M (SD)**	***F*(1,61)**	**1**	**2**	**3**	**4**	**5**	**6**

1. GL	/	/	/						
2. Prevention focus	4.670 (0.610)	4.542 (0.785)	0.525	–0.092					
3. Learning orientation	5.360 (0.758)	5.034 (0.636)	3.382	–0.229	0.146				
4. Gender	0.290 (0.461)	0.563 (0.504)	4.989*	0.275*	–0.082	–0.134			
5. Age	20.645 (0.798)	20.719 (0.772)	0.138	0.048	0.006	–0.095	0.065		
6. Trait anxiety	4.032 (1.402)	3.875 (1.519)	0.182	–0.055	–0.017	–0.152	0.162	0.143	

^a^N = 61 (in experiment 1a) and N = 63 (in experiment 1b). ^b^Leaders’ encourage-to-explore behaviors (EE): 0 = Less encourage-to-explore behaviors (LEE); 1 = More encourage-to-explore behaviors (MEE); Leaders’ guide-to-learn behaviors (GL): 0 = Less guide-to-learn behaviors (LGL); 1 = More guide-to-learn behaviors (MGL). ^c^Gender: 1 = Male; 0 = Female. ^d^*p < 0.05, ***p < 0.001.

Hypothesis 1 proposes that leaders’ encourage-to-explore behaviors are positively related to employee learning orientation. We ran an analysis of variance (ANOVA) in SPSS22.0 with the two encourage-to-explore behavior experimental conditions (more encourage-to-explore behavior vs. less encourage-to-explore behavior) as independent variables and learning orientation as the dependent variable. Results demonstrated a significant effect [*F*(1,59) = 4.792, *p* < 0.05]. Respondents under more leaders’ encourage-to-explore behavior reported higher learning orientation (M_*LEE*_ = 5.228, M_*M*__*EE*_ = 5.560)^1^. This, thus, supported Hypothesis 1.

To test Hypothesis 3, we conducted a 2 × 2 analysis of covariance (ANCOVA) in SPSS22.0 with the two encourage-to-explore behavior experimental conditions and two promotion focus conditions (low level of promotion focus coded as 0 vs. high level of promotion focus coded as 1) as independent variables, control variables (gender, age, and trait anxiety) as co-variables, and learning orientation as the dependent variable. The results are shown in Table 2. As shown in the table, the moderating effect of promotion focus on the relationship between encourage-to-explore behavior and learning orientation is significant [*F*(1, 54) = 9.320, $\eta_{p}^{2}=0.147$, *p* < 0.01]. Specifically, for individuals with low level of promotion focus, when compared to the effect of less encourage-to-explore behaviors (*N* = 15, *M* = 4.890, *SD* = 0.705), more encourage-to-explore behaviors induce greater learning orientation (*N* = 15, *M* = 5.600, *SD* = 0.499) was seen. However, for individuals with high level of promotion focus, the effect of more encourage-to-explore behaviors on learning orientation (*N* = 16, *M* = 5.520, SD = 0.354) yield no significant difference from the effect of less encourage-to-explore behaviors (*N* = 15, *M* = 5.736, SD = 0.379).

**TABLE 2:** Results of analysis of covariance (Study 1).

Variables		Learning Orientation					
		Experiment 1a			Experiment 1b		
		*F*	*df*	$\eta_{p}^{2}$	*F*	Df	$\eta_{p}^{2}$
Co-variables	Gender	2.016	1, 54	0.036	0.270	1,56	0.005
	Age	1.779	1, 54	0.032	0.166	1,56	0.003
	Anxiety	3.283^+^	1, 54	0.057	1.125	1,56	0.020
IV	Encourage-to-explore	3.312^+^	1, 54	0.058			
	Promotion focus	9.464**	1, 54	0.149			
	Guide-to-learn				3.042^+^	1,56	0.039
Interaction	Prevention focus				2.285	1,56	0.052
	EE × Promotion focus	9.320**	1, 54	0.147			
	GL × Prevention focus				3.542^+^	1,56	0.059

^a^N = 61 (in experiment 1a) and N = 63 (in experiment 1b). ^b^Leaders’ encourage-to-explore behaviors (EE): 0 = Less encourage-to-explore behaviors (LEE); 1 = More encourage-to-explore behaviors (MEE); Leaders’ guide-to-learn behaviors (GL): 0 = Less guide-to-learn behaviors (LGL); 1 = More guide-to-learn behaviors (MGL). Promotion focus: 0 = low level of promotion focus; 1 = high level of promotion focus. Prevention focus: 0 = low level of prevention focus; 1 = high level of prevention focus. ^c^Gender: 1 = Male; 0 = Female. ^d +^p < 0.1, **p < 0.01.

*Post hoc* comparisons were also conducted by applying the *Bonferroni* method. Results demonstrated that there was a significant difference between low-promotion/low encourage-to-explore condition and low-promotion/high encourage-to-explore condition (*mean difference* = −0.711, SE = 0.233, *P* < 0.01, 95%CI = [−1.345, −0.077]).

**FIGURE 3:**
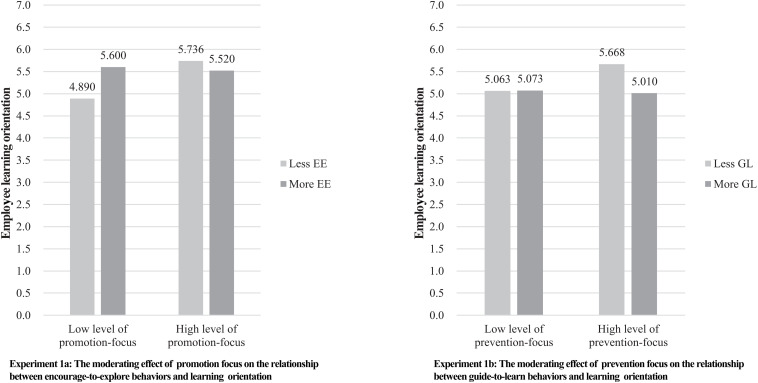
The moderating effect of regulatory focus (Study 1).

Figure 3 depicts the moderating effect. This thus supported Hypothesis 3, which proposes that the positive association between encourage-to-explore behaviors and employee learning will be stronger for employees with the low level of promotion focus.

In experiment 1b, we aim to verify the interactive effect between guide-to-learn behaviors and prevention focus on learning orientation. Results of ANOVA indicated that respondents supervised by more guide-to-learn behaviors reported less learning orientation than those who were supervised by less guide-to-learn behaviors (M_*L*__*GL*_ = 4.670, M_*M*__*GL*_ = 4.542)^2^. The effect was slightly significant [*F*(1,61) = 3.382, *p* < 0.1]. Additionally, as shown in Table 2, the moderating effect of prevention focus on the relationship between guide-to-learn behaviors and learning orientation is slightly significant [*F*(1, 56) = 3.542, $\eta_{p}^{2}=0.059$, *p* < 0.1]. Specifically, as shown in Figure 3, for individuals with low level of prevention focus, the effect of more guide-to-learn behaviors on learning orientation (*N* = 16, *M* = 5.063, SD = 0.637) showed less difference from the effect of less guide-to-learn behaviors (*N* = 16, *M* = 5.073, SD = 0.696).

While for individuals with high level of prevention focus, more guide-to-learn behaviors proved to induce less learning orientation (*N* = 16, *M* = 5.010, SD = 0.654) than less guide-to-learn behaviors (*N* = 15, *M* = 5.668, SD = 0.718). This partially supported Hypothesis 4, which proposes that when the level of prevention focus is high, there is a negative association between guide-to-learn behaviors and employee learning orientation. The results of *post hoc* comparisons showed that high-prevention/low guide-to-learn condition was significantly different from high-prevention/high guide-to-learn condition (*mean difference* = 0.772, *SE* = 0.228, *P* < 0.01, 95%CI = [−1.392, −0.153]).

In study 1, we examined our hypotheses with two experiments. While Hypotheses 1 and 3 were supported, H2 was not supported and H4 was partially supported. As it is difficult to manipulate low, moderate, and high level of guide-to-learn behaviors in the experiment, the proposed inverted U-shaped relationship between leaders’ guide-to-learn behaviors and employee learning orientation should be verified by other methods.

### Study 2

To further validate the results of Study 1 in the workplace context and test hypotheses, which are difficult to be verified by experiment design, we conduct another study (Study 2) with paired data from working employees and their supervisors.

#### Sample and Procedure

The sample of study 2 included 334 pairs of employees and their direct supervisors in 22 departments at a large state-owned enterprise in China. Overall, the participants represent hundreds of branches and departments in all parts of China. The mean age of employees was 41.63 years (SD = 7.68), with ages ranging from 20 to 58 years. Females constituted 38% of the participants and 62% were males. The average work experience of the employees was 20.62 years. The average age of leaders was 46.52. Among the leaders, 28.38% were female and 71.62% were male.

Paper copies of the study’s questionnaires were distributed separately to the employees and their direct supervisors through the department chiefs. We encoded each piece of the questionnaires in envelopes and asked the department chiefs to deliver them in pairs according to the codes. First, surveys were distributed to 350 supervisors asking them to evaluate their frequent coaching behaviors. Then about a week later, the department chiefs withdrew previous questionnaires and gave other surveys to the followers. The independent-variable questionnaires were distributed to supervisors and the dependent-variable questionnaires were distributed to followers. Before distributing the survey instrument, the original scales were translated into Chinese and then back translated into English by two bilingual speakers to check the accuracy of the translation (Brislin, 1986).

#### Measures

##### Leaders’ Coaching Behaviors

The items measuring coaching behaviors were derived from the study of Naveh et al. (2015), and leaders were asked to access the measurement of their coaching behaviors, six items for encourage-to-explore and 5 items for guide-to-learn, respectively. The leaders responded to the items using a seven-point Likert-type scale (1 = “strongly disagree”; 7 = “strongly agree”). The α-coefficients for encourage-to-explore behaviors and guide-to-learn behaviors were 0.930 and 0.962, respectively.

##### Regulatory Focus of Employees

Participants were instructed to rate their regulatory focus on the same scale in study 1 (Lockwood et al., 2002). The participants responded to the items using a seven-point Likert-type scale (1 = “strongly disagree”; 7 = “strongly agree”). The α-coefficients for promotion focus and prevention focus were 0.860 and 0.834, respectively.

##### Employee Learning Orientation

We adopted Gong et al. (2009) six-item scale to assess individual learning orientation at the work setting. The participants responded to the items using a seven-point Likert-type scale (1 = “strongly disagree”; 7 = “strongly agree”). The α-coefficient for employee learning orientation was 0.919.

##### Control Variables

The following control variables were used in study 2: (a) gender, (b) age (in years), and (c) trait anxiety, which controlled the level of emotional control.

#### Confirmatory Factor Analyses

Before testing our hypotheses, we conducted a confirmatory factor analysis (CFA) using AMOS 22.0 with maximum likelihood estimations to examine the measurement model fit and common method bias. We tested the model that consisted of five factors, including encourage-to-explore behaviors (six items), guide-to-learn behaviors (five items), promotion focus of employee (nine items), prevention focus of employee (nine items), and employee learning orientation (six items). Results showed that the expected five-factor model fit the data well (χ*^2^* = 0.66, df = 497, χ*^2^*/df = 3.43, NNFI = 0.92, CFI = 0.87, IFI = 0.87, RMSEA = 0.08). All the standardized factor loadings in the model were above 0.50 (the majority of the loadings were above 0.70). Additionally, given that we collected data from a single source, our data are susceptible to common source bias. Following the recommendation by Podsakoff et al. (2003), we applied the single unmeasured latent method. Results of one-factor model in which all items were allowed to load on one factor showed a poor fit level (χ*^2^* = 5,842.09, df = 487, χ*^2^*/df = 12.00, NNFI = 0.41, CFI = 0.40, IFI = 0.40, RMSEA = 0.18). Since the expected five-factor model yielded better fit, the common method bias was not a severe problem in our study.

#### Results

Table 3 presents the means, standard deviations, and correlations among the variables in this study.

**TABLE 3:** Means, standard deviations, and correlations (Study 2).

	*M*	SD	1	2	3	4	5	6	7	8
1. Gender	0.620	0.486								
2. Age	41.632	7.679	–0.087							
3. Anxiety	3.246	1.495	0.145**	−0.113*						
4. Encourage-to-explore	4.583	1.149	−0.178**	–0.031	–0.089					
5. Guide-to-learn	5.667	0.771	−0.187**	0.098	–0.146	0.319**				
6. Promotion focus	4.315	0.961	0.028	−0.150**	0.119*	0.111*	0.006			
7. Prevention focus	4.244	0.977	0.120*	–0.055	0.244**	–0.010	–0.090	0.707*		
8. Employee learning orientation	5.150	0.973	–0.014	−0.122*	–0.029	0.135*	0.033	0.229**	0.420**	

N = 334; ^+^p < 0.1; *p < 0.05; **p < 0.01.

In study 2, regressions were conducted to test the hypotheses using SPSS 22.0. The results were shown in Table 4. Each variable was centered for product terms before computing the multiplicative terms. To test Hypothesis 1, we regressed supervisor-rated encourage-to-explore behaviors on employee-rated employee learning orientation. As Hypothesis 1 proposed, leaders’ encourage-to-explore behaviors had a positive relationship with employee learning orientation (β = 0.109, *p* < 0.05), shown in Model 2 (Table 4). Hypothesis 1 was supported: leaders’ exploration-encouraging behaviors can improve followers’ learning orientation. To test Hypothesis 2, we regressed employee learning orientation on control variables, guide-to-learn behaviors squared, the interaction between leaders’ guide-to-learn behaviors and employees’ prevention focus, and the interaction between leaders’ guide-to-learn behaviors squared and employees’ prevention focus. As Hypothesis 2 proposed, leaders’ guide-to-learn behaviors squared had a negative and significant relationship with employee learning orientation (β = −0.109, *p* < 0.01), shown in Model 4 (Table 4). Hypothesis 2 was supported: leaders’ guide-to-learn behaviors and employee learning orientation showed an inverted U-shaped relationship, with the highest level of employee learning orientation at intermediate levels of the leader’s guiding behaviors.

**TABLE 4:** Results of regression analyses of coaching behaviors on employee learning (Study 2).

DV		Employee learning orientation					
		Model 1	Model 2	Model 3	Model 4	Model 5	Model 6
Control	Gender	−0.039 (0.111)	0.005 (0.112)	−0.019 (0.853)	−0.039 (0.111)	−0.098 (0.108)	−0.040 (0.100)
	Age	−0.016* (0.007)	−0.015* (0.007)	−0.008 (0.006)	−0.016* (0.007)	−0.016* (0.007)	−0.007 (0.006)
	Anxiety	−0.026 (0.036)	−0.020 (0.036)	−0.047 (0.033)	−0.026 (0.036)	−0.070* (0.036)	−0.042 (0.033)
IV	Encourage-to-explore behaviors (EE)		0.109* (0.047)	0.061 (0.043)			0.117* (0.047)
	Guide-to-learn behaviors (GL)				1.153* (0.462)	1.574** (0.452)	1.763*** (0.440)
	GL^2^				−0.109* (0.045)	−0.145** (0.044)	−0.173*** (0.044)
Moderator	Promotion focus			0.413*** (0.051)			0.449*** (0.071)
	Prevention focus					0.279*** (0.056)	−0.037 (0.072)
	EE × Promotion focus			−0.106* (0.047)			−0.102* (0.047)
	GL × Prevention focus					−2.544** (0.800)	−2.635*** (0.745)
	GL^2^ × Prevention focus					0.229** (0.078)	0.247*** (0.073)
Df		3, 330	4, 329	6, 327	5, 328	8, 325	11, 322
*F*		1.905	2.805*	14.104**	2.432*	6.474**	10.615**
*AdjR*^2^		0.008	0.021	0.191	0.021	0.116	0.241
△*R*^2^			0.013*	0.182**	0.013*	0.108**	0.233**

N = 334; Unstandardized coefficients are shown with standard errors in parentheses. ^+^p < 0.1; **p < 0.01.

**FIGURE 4:**
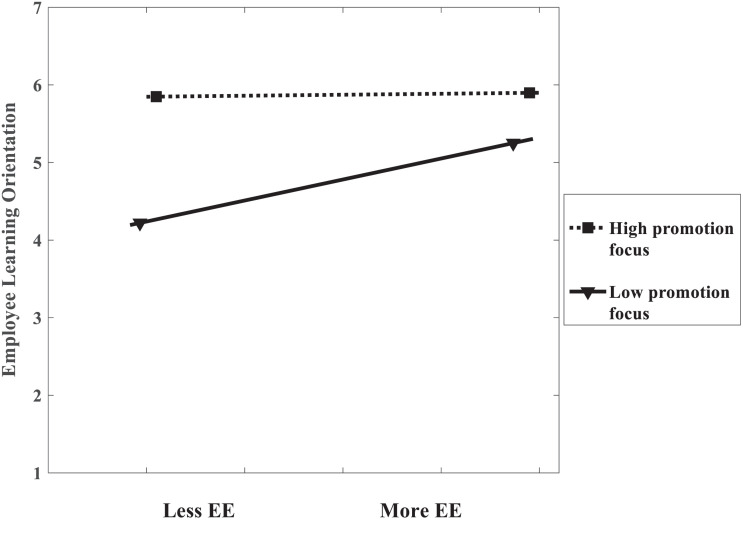
The moderating effect of promotion focus (Study 2). Note: High refers to 1SD above the mean; and Low refers to 1SD below the mean. EE refers to encourage-to-explore behaviors.

Hypothesis 3 proposed that employees’ promotion focus would moderate the association between encourage-to-explore behaviors and employee learning orientation. In Model 3 (Table 4), the multiplicative term between encourage-to-explore behaviors and promotion focus was introduced into the regression equation, and the product term significantly and negatively associated with the employee learning orientation (β = −0.106, *p* < 0.05). Hypothesis 3 was thus supported. Consistent with the results of the moderation effects, a simple slopes analysis showed that the association between leaders’ encourage-to-explore behaviors and employee learning orientation was positive and significant in the low promotion focus group (1SD below the mean, *simple slope* = 0.167, *t* = 2.709, *p* < 0.01), but the association was non-significant in the high promotion focus group (1SD above the mean, *simple slope* = −0.045, *t* = −0.694, *p* > 0.1), shown in the plotted figure (Figure 4).

Hypothesis 4 proposed that employee prevention focus positively moderates the relationship between leaders’ guide-to-learn behaviors and employee learning orientation. In Model 5 (Table 4), the interaction between prevention focus and guide-to-learn behaviors squared was significantly and positively associated with employee learning orientation (β = 0.229, *p* < 0.01). Hypothesis 4 was thus supported.

**FIGURE 5:**
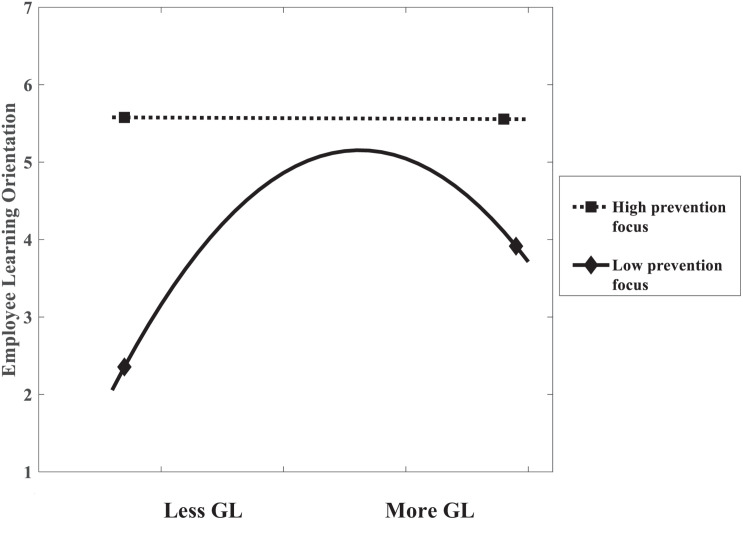
The moderating effect of prevention focus (Study 2). Note: High refers to 1SD above the mean; and Low refers to 1SD below the mean. GL refers to guide-to-learn behaviors.

Figure 5 illustrates that the curvilinear relationship between leaders’ guide-to-learn behaviors and employee learning orientation depended on the level of employees’ prevention focus. When an individual’s level of prevention focus was high (1SD above the mean), the association between guide-to-learn behaviors and employee learning orientation was not significant (β = −0.028, ns), and the association between guide-to-learn behaviors squared and employee learning orientation was not significant (β = −0.063, ns) as well. When the individual’s level of prevention focus was low (1SD below the mean), leaders’ guide-to-learn behaviors squared had a negative and significant relationship with employee learning orientation (β = −0.552, *p* < 0.01) and there was an inverted U-shaped relationship between leaders’ guide-to-learn behaviors and employee learning orientation.

## Discussion

### Theoretical Contributions

This study makes several significant contributions to the field. First, by identifying two styles of leaders’ coaching behaviors and examine their effects separately, this study contributed to the literature on leadership. Previous studies have demonstrated the positive associations between general leadership styles and employee learning, including transformational leadership (Chi and Huang, 2014), authentic leadership (Mehmood and Hamstra, 2016), servant leadership (Tang et al., 2016), as well as coaching (Matsuo, 2018). The results of this study showed that leader’s encourage-to-explore and guide-to-learn behaviors have effects on employee learning orientation, which further supported existing findings. Moreover, our findings have filled in the research gap on the functions of different and specific type of coaching behaviors (Kim et al., 2013; Steelman and Wolfeld, 2018). Specifically, we identified two types of coaching behaviors including encourage-to-explore and guide-to-learn behaviors. Results demonstrated that encourage-to-explore behaviors promote employees’ learning orientation, while guide-to-learn behaviors showed an inverted U-shaped relationship with employees’ learning orientation. Once above a certain level, guidance from leaders does more to undermine employees learning willingness rather than promoting them. These results thus enrich current leadership literature by examining how specific type of coaching behaviors affect employee outcomes.

Second, this study extends the research of active learning by emphasizing the role of leaders in learning interventions. The active learning literature has demonstrated that learning interventions, such as exploratory learning (McDaniel and Schlager, 1990), error management training (Keith and Frese, 2008), and guided exploration (Bell and Kozlowski, 2002) enhance important learning outcomes. However, most of them have commonly explored the effect of learning interventions in a broad environment, ignoring the role of leaders playing in these interventions. This study expands this by investigating how leaders carrying out learning interventions, including encourage-to-explore and guide-to-learn behaviors. Results show that while encourage-to-explore behaviors always produce good outcomes, guide-to-learn behaviors demonstrated the “Too-much-of-a-Good-Thing (TMGT)” effect (Sharma and Kirkman, 2015). This finding provides further support that although active learning approaches commonly benefit trainees, it not always associated with better outcomes (Bell and Kozlowski, 2008).

Third, by exploring the moderating role of regulatory focus in the relationship between leaders’ coaching behaviors and employee learning orientation, our research contributed to the research on complementary fit. Results showed that for employees with low promotion focus, more encourage-to-explore behaviors induce greater learning orientation; while for employees who are highly promotion focused, more encourage-to-explore behaviors yield no significant difference. This is consistent with previous studies of complementary fit (e.g., Kausel and Slaughter, 2011; Ehrhardt and Ragins, 2019). Moreover, we also extend existing studies of complementary fit to the curvilinear perspective. This study revealed that when employees have low level of prevention focus, there was an inverted U-shaped relationship between leaders’ guide-to-learn behaviors and employee learning orientation. Since the curvilinear effect of complementary fit has not been proved previously, our paper shed light on the importance to investigate the boundary conditions of complementary fit.

### Practical Implications

We have shown the importance of employee learning orientation for both individuals and organizations, and it is of great practical value to examine their facilitators in the workplace. Our results imply that managers need to reap the benefits of employee learning by adopting appropriate coaching styles. We proved that both encourage-to-explore behaviors and guide-to-learn behaviors would facilitate employees’ learning orientation. Therefore, for general managers who are not able to provide specific guidance to employees due to lack of specialized skills, encourage-to-explore can be considered as an alternative way to promote employees’ learning orientation. If managers are capable of guiding employees to learn, they should know that too much guiding behaviors may also be detrimental to employee learning orientation. One good way is to exhibit guide-to-learn coaching with a certain level. Additionally, employees’ regulatory focus should be considered as well during the coaching process. Since employees with higher levels of regulatory focus cannot be easily affected by leaders’ coaching behaviors, a more desirable way to encourage their learning is to provide a relaxed environment and sufficient support. Managers can focus more on employees with lower levels of regulatory focus, with whom their learning motivation can be significantly promoted by coaching behaviors. Specifically, for employees with lower levels of promotion focus, managers can provide more encouragements to exploratory activities. Whereas for employees with lower levels of prevention focus, proper guidance is beneficial for learning, but too many guiding behaviors may result in employees’ dependence on leaders and loss of their learning autonomy. To summarize, leaders’ coaching behaviors may increase employees’ learning orientation, depending on the styles and levels of coaching behaviors, as well as on employees’ regulatory focus.

### Limitations and Directions for Future Research

This study contributes to the research in the field of coaching behaviors and employee learning, but it also has some potential limitations in several aspects. First, there were some concerns regarding the methodology and data. Specifically, we measured leaders’ learning promotion behaviors and employee learning by self-report scales in study 2, as we aimed to discover how leaders’ intentions to promote employee learning affect employees’ learning orientation. The multi-source design enables us to alleviate the common method bias. However, our temporally lagged design cannot rule out the possibility that the learning orientation of employees brings out different leader behaviors. Additionally, although we designed an experiment to clarify the causality in study 1, the U-shaped effects have not been proved and the undergraduate sample limits the generality of the findings. Therefore, we encourage future research to test this hypothesis more rigorously through multiple time points for measurement or field experiments. A longitudinal design could also be conducted to strengthen the possibility of inferring causality. When addressing the concern for data, another limitation was our limited samples and departments. Collecting data about workplace learning orientation and learning behavior in organizations is challenging, and researchers are still working on increasing the reliability and validity of existing measures (Naveh et al., 2015). Future research may compare the relationship between coaching behavior and learning across the professional career, adding more types of industries and jobs to the sample.

Second, the difference between leaders’ coaching behaviors and active learning environments was not considered in this study, as we did not collect data at the team level to assess shared climate perceptions. To avoid the practice effect or fatigue effect, we did not measure the learning orientation of participants before the manipulation in study 1, so control groups could be set for comparison in future studies. For future research, it would be worthwhile to explore how leaders’ intentions to promote employee learning affect actual employee learning behaviors, which can be assessed by other colleagues.

Third, although we proposed that individual regulatory focus may moderate the relationship between leaders’ coaching behavior and employees’ learning orientation, we just focused on two conditions: (a) the interaction between leaders’ encourage-to-explore behaviors and employees’ promotion focus, and (b) the interaction between leaders’ guide-to-learn behaviors and employees’ prevention focus. We chose to explore these two conditions because based on prior findings, employees with promotion focus may typically have a better regulatory fit with the leader who provides a promotion-oriented coaching manner, while employees with prevention focus may typically experience a better regulatory fit with the leader who provides a prevention-oriented coaching manner (Sue-Chan et al., 2012; Lin et al., 2017). Encourage-to-explore behaviors encourage explorative activities and novel working approaches, which is a typically promotion-oriented coaching manner (Righetti et al., 2011); while guide-to-learn behaviors has been shown to prime a prevention orientation which aims to avoid failure during employees’ work process (Hong and Lee, 2008; Sue-Chan et al., 2012). Therefore, we chose to investigate how regulatory fit affect employees in this study. We didn’t examine the mechanism on how regulatory mismatch affects employee learning, including (c) encourage-to-explore behavior and employee prevention focus; and (d) guide-to-learn behavior and employee promotion focus. Future studies are encouraged to explore these two conditions as well.

Finally, we did not explore the internal mechanism in the association between leader behaviors and employee learning orientation. Leaders’ coaching behaviors can encourage employees to learn through exceeding their intrinsic motivation or specific emotions, and therefore, different leader behaviors may work on learning orientation in different ways. An interesting direction for empirical research is to discuss the procedural mechanism by which leaders’ coaching behaviors impact employees’ learning orientation.

## Conclusion

We theoretically proposed and empirically examined the associations between leaders’ coaching behaviors and employee learning orientation, which were moderated by employees’ regulatory focus. The findings of this study demonstrated that leaders’ encourage-to-explore behaviors and guide-to-learn behaviors have different impacts on employee learning orientation, and employees’ promotion- and prevention-regulatory foci have different moderating effects on the relationships. Spcifically, encourage-to-explore behaviors were positively related to employee learning orientation. Guide-to-learn behaviors and employee learning orientation had an inverted U-shape relationship. When employees’ level of promotion focus was low, encourage-to-explore behaviors were positively and significantly related to employee learning orientation. When employees’ level of prevention focus was low, there was a significant curvilinear (inverted U-shaped) relationship between guide-to-learn behaviors and employee learning orientation.

## Data Availability Statement

The datasets generated for this study are available on request to the corresponding author.

## Ethics Statement

The studies involving human participants were reviewed and approved by Central University of Finance and Economics. The patients/participants provided their written informed consent to participate in this study.

## Author Contributions

WL mainly led the research design, data collection, data analysis, and manuscript drafting. STX mainly led the literature review, hypotheses developing, data analysis, experiment, and manuscript revision. Both authors contributed to the article and approved the submitted version.

## Conflict of Interest

The authors declare that the research was conducted in the absence of any commercial or financial relationships that could be construed as a potential conflict of interest.

## Footnotes

1 LEE means less encourage-to-explore behaviors; MEE means more encourage-to-explore behaviors.

2 LGL means less guide-to-learn behaviors; MGL means more guide-to-learn behaviors.

## References

[B1] Agarwal R. Angst C. M. Magni M. (2009). The performance effects of coaching: a multilevel analysis using hierarchical linear modeling. *Int. J. Hum. Resour. Manag.* 20 2110–2134. 10.1080/09585190903178054

[B2] Ames C. Archer J. (1988). Achievement goals in the classroom: students’ learning strategies and motivation processes. *J. Educ. Psychol.* 80 260–267. 10.1037/0022-0663.80.3.260

[B3] Atkinson J. W. (1964). *An Introduction to Motivation.* Princeton, NJ: Van Nostrand.

[B4] Bakker A. B. Demerouti E. Brummelhuis L. L. T. (2012). Work engagement, performance, and active learning: the role of conscientiousness. *J. Vocat. Behav.* 80 555–564. 10.1016/j.jvb.2011.08.008

[B5] Bell B. S. Kozlowski S. W. J. (2002). Adaptive guidance: enhancing self-regulation, knowledge, and performance in technology-based training. *Pers. Psychol.* 55 267–306. 10.1111/j.1744-6570.2002.tb00111.x

[B6] Bell B. S. Kozlowski S. W. J. (2008). Active learning: effects of core training design elements on self-regulatory processes, learning, and adaptability. *J. Appl. Psychol.* 93 296–316. 10.1037/0021-9010.93.2.296 18361633

[B7] Bell B. S. Kozlowski S. W. J. (2010). “Toward a theory of learner-centered training design: an integrative framework of active learning,” in *Learning, Training, and Development in Organizations*, eds Kozlowski S. W. J. Salas E., (New York, NY: Routledge), 263–302.

[B8] Bezuijen X. M. Berg P. T. V. D. Dam K. V. Thierry H. (2009). Pygmalion and employee learning: the role of leader behaviors. *J. Manag.* 35 1248–1267. 10.1177/0149206308329966

[B9] Bezuijen X. M. Dam K. V. Berg P. T. V. D. Thierry H. (2010). How leaders stimulate employee learning: a leader–member exchange approach. *J. Occup. Organ. Psychol.* 83 673–692. 10.1348/096317909x468099 30467716

[B10] Brett J. F. VandeWalle D. (1999). Goal orientation and goal content as predictors of performance in a training program. *J. Appl. Psychol.* 84 863–873. 10.1037/0021-9010.84.6.863

[B11] Brislin R. W. (1986). “The wording and translation of research instruments,” in *Field Method in Cross-Cultural Research*, eds Lonner W. J. Berry J. W., (Beverly Hills, CA: Sage), 137–164.

[B12] Brockner J. Higgins E. T. (2001). Regulatory focus theory: implications for the study of emotions at work. *Organ. Behav. Hum. Decis. Process.* 86 35–66. 10.1006/obhd.2001.2972

[B13] Button S. B. Mathieu J. E. Zajac D. M. (1996). Goal orientation in organizational research: a conceptual and empirical foundation. *Organ. Behav. Hum. Decis. Process.* 67 26–48. 10.1006/obhd.1996.0063

[B14] Chi N. W. Huang J. C. (2014). Mechanisms linking transformational leadership and team performance: the mediating roles of team goal orientation and group affective tone. *Group Organ. Manag.* 39 300–325. 10.1177/1059601114522321

[B15] Christie A. Barling J. Turner N. (2011). Pseudo-transformational leadership: model specification and outcomes. *J. Appl. Soc. Psychol.* 41 2943–2984. 10.1111/j.1559-1816.2011.00858.x

[B16] Coetzer A. (2007). Employee perceptions of their workplaces as learning environments. *J. Workplace Learn.* 19 417–434. 10.1108/13665620710819375

[B17] Dahling J. J. Taylor S. R. Chau S. L. Dwight S. A. (2016). Does coaching matter? A multilevel model linking managerial coaching skill and frequency to sales goal attainment. *Pers. Psychol.* 69 863–894. 10.1111/peps.12123

[B18] Debicki B. J. Kellermanns F. W. Barnett T. Pearson A. W. Pearson R. A. (2016). Beyond the big five: the mediating role of goal orientation in the relationship between core self-evaluations and academic performance. *Int. J. Manag. Educ.* 14 273–285. 10.1016/j.ijme.2016.05.002

[B19] Debowski S. Wood R. E. Bandura A. (2001). Impact of guided exploration and enactive exploration on self-regulatory mechanisms and information acquisition through electronic search. *J. Appl. Psychol.* 86 1129–1141. 10.1037/0021-9010.86.6.1129 11768056

[B20] Deci E. L. Ryan R. M. (1980). “The empirical exploration of intrinsic motivation processes,” in *Advances in Experimental Social Psychology*, Vol. 13 ed. Berkowitz L., (New York, NY: Academic Press), 39–80. 10.1016/s0065-2601(08)60130-6

[B21] Dragoni L. (2005). Understanding the emergence of state goal orientation in organizational work groups: the role of leadership and multilevel climate perceptions. *J. Appl. Psychol.* 90 1084–1095. 10.1037/0021-9010.90.6.1084 16316267

[B22] Dweck C. S. (1986). Motivational processes affecting learning. *Am. Psychol.* 41 1040–1048. 10.1037/0003-066x.41.10.1040

[B23] Dweck C. S. (2000). *Self-Theories: Their Role in Motivation, Personality, and Development.* Philadelphia, PA: Psychological Press.

[B24] Dweck C. S. Leggett E. L. (1988). A social-cognitive approach to motivation and personality. *Psychol. Rev.* 95 256–273. 10.1037/0033-295x.95.2.256

[B25] Edwards J. R. (1996). An examination of competing versions of the person-environment fit approach to stress. *Acad. Manag. J.* 39 292–339. 10.2307/256782

[B26] Ehrhardt K. Ragins B. R. (2019). Relational attachment at work: a complementary fit perspective on the role of relationships in organizational life. *Acad. Manag. J.* 62 248–282. 10.5465/amj.2016.0245

[B27] Ellinger A. D. Hamlin R. G. Beattie R. S. Wang Y. L. Mcvicar O. (2011). “Managerial coaching as a workplace learning strategy,” in *Supporting Workplace Learning*, Vol. 5 eds Poell R. van Woerkom M., (Dordrecht: Springer Netherlands), 71–87. 10.1007/978-90-481-9109-3_5

[B28] Förster J. Friedman R. S. Liberman N. (2004). Temporal construal effects on abstract and concrete thinking: consequences for insight and creative cognition. *J. Pers. Soc. Psychol.* 87 177–189. 10.1037/0022-3514.87.2.177 15301626

[B29] Friedman R. S. Förster J. (2001). The effects of promotion and prevention cues on creativity. *J. Pers. Soc. Psychol.* 81 1001–1013. 10.1037/0022-3514.81.6.1001 11761303

[B30] Gagné M. Deci E. L. (2005). Self-determination theory and work motivation. *J. Organ. Behav.* 26 331–362. 10.1002/job.322

[B31] Gong Y. Huang J. C. Farh J. L. (2009). Employee learning orientation, transformational leadership, and employee creativity: the mediating role of employee creative self-efficacy. *Acad. Manag. J.* 52 765–778. 10.5465/amj.2009.43670890

[B32] Greguras G. J. Diefendorff J. M. (2009). Different fits satisfy different needs: linking person-environment fit to employee commitment and performance using self-determination theory. *J. Appl. Psychol.* 94 465–477. 10.1037/a0014068 19271801

[B33] Gully S. M. Chen G. (2010). “Individual differences, attribute–treatment interactions, and training outcomes,” in *Learning, Training, and Development in Organizations*, eds Kozlowski S. W. J. Salas E., (New York, NY: Routledge), 3–64.

[B34] Harrison R. V. (1978). “Person–environment fit and job stress,” in *Stress at Work*, eds Cooper C. L. Payne R., (New York, NY: Wiley), 175–205.

[B35] Heslin P. A. Vandewalle D. Latham G. P. (2006). Keen to help? managers’ implicit person theories and their subsequent employee coaching. *Pers. Psychol.* 59 871–902. 10.1111/j.1744-6570.2006.00057.x

[B36] Heyman G. D. Dweck C. S. (1992). Achievement goals and intrinsic motivation: their relation and their role in adaptive motivation. *Motiv. Emot.* 16 231–247. 10.1007/bf00991653

[B37] Higgins E. T. (1997). Beyond pleasure and pain. *Am. Psychol.* 52 1280–1300. 10.1037/0003-066x.52.12.1280 9414606

[B38] Higgins E. T. (1998). “Promotion and prevention: regulatory focus as a motivational principle,” in *Advances in Experimental Social Psychology*, Vol. 30 ed. Zanna M. P., (New York, NY: Academic Press), 1–46. 10.1016/s0065-2601(08)60381-0

[B39] Higgins E. T. Tykocinski O. (1992). Self-discrepancies and biographical memory: personality and cognition at the level of psychological situation. *Pers. Soc. Psychol. Bull.* 18 527–535. 10.1177/0146167292185002

[B40] Hong J. Lee A. Y. (2008). Be fit and be strong: mastering self-regulation through regulatory fit. *J. Consum. Res.* 34 682–695. 10.1086/521902

[B41] Jones R. J. Woods S. A. Guillaume Y. R. (2016). The effectiveness of workplace coaching: a meta-analysis of learning and performance outcomes from coaching. *J. Occup. Organ. Psychol.* 89 249–277. 10.1111/joop.12119

[B42] Kark R. Van-Dijk D. (2007). Motivation to lead, motivation to follow: the role of the self-regulatory focus in leadership processes. *Acad. Manag. Rev.* 32 500–528. 10.5465/amr.2007.24351846

[B43] Kark R. Katz-Navon T. Delegach M. (2015). The dual effects of leading for safety: the mediating role of employee regulatory focus. *J. Appl. Psychol.* 100 1332–1348. 10.1037/a0038818 25664472

[B44] Kausel E. E. Slaughter J. E. (2011). Narrow personality traits and organizational attraction: evidence for the complementary hypothesis. *Organ. Behav. Hum. Decis. Process.* 114 3–14. 10.1016/j.obhdp.2010.08.002

[B45] Keith N. Frese M. (2005). Self-regulation in error management training: emotion control and metacognition as mediators of performance effects. *J. Appl. Psychol.* 90 677–691. 10.1037/0021-9010.90.4.677 16060786

[B46] Keith N. Frese M. (2008). Effectiveness of error management training: a meta-analysis. *J. Appl. Psychol.* 93 59–69. 10.1037/0021-9010.93.1.59 18211135

[B47] Kim S. Egan T. M. Kim W. Kim J. (2013). The impact of managerial coaching behavior on employee work-related reactions. *J. Bus. Psychol.* 28 315–330. 10.1007/s10869-013-9286-9

[B48] Kirmani A. Zhu R. (2007). Vigilant against manipulation: the effect of regulatory focus on the use of persuasion knowledge. *J. Mark. Res.* 44 688–701. 10.1509/jmkr.44.4.688 11670861

[B49] Kluger A. N. Denisi A. (1996). The effects of feedback interventions on performance: a historical review, a meta-analysis, and a preliminary feedback intervention theory. *Psychol. Bull.* 119 254–284. 10.1037/0033-2909.119.2.254

[B50] Kozlowski S. W. Gully S. M. Brown K. G. Salas E. Smith E. M. Nason E. R. (2001). Effects of training goals and goal orientation traits on multidimensional training outcomes and performance adaptability. *Organ. Behav. Hum. Decis. Process.* 85 1–31. 10.1006/obhd.2000.2930 11341815

[B51] Kristof A. L. (1996). Person-organization fit: an integrative review of its conceptualizations, measurement, and implications. *Pers. Psychol.* 49 1–49. 10.1111/j.1744-6570.1996.tb01790.x

[B52] Leung K. Chen T. Chen G. (2012). Learning goal orientation and creative performance: the mediating roles of intrinsic motivation. *Acad. Manag. Annu. Meet. Proc.* 2012 1247–1252.

[B53] Liem A. D. Lau S. Nie Y. (2008). The role of self-efficacy, task value, and achievement goals in predicting learning strategies, task disengagement, peer relationship, and achievement outcome. *Contemp. Educ. Psychol.* 33 486–512. 10.1016/j.cedpsych.2007.08.001

[B54] Lin W. J. Lin C. Y. Chang Y. H. (2017). The impact of coaching orientation on subordinate performance: the moderating effects of implicit person theory and LMX. *Asia Pac. J. Hum. Resour.* 55 86–105. 10.1111/1744-7941.12107

[B55] Lockwood P. Jordan C. H. Kunda Z. (2002). Motivation by positive or negative role models: regulatory focus determines who will best inspire us. *J. Pers. Soc. Psychol.* 83 854–864. 10.1037/0022-3514.83.4.854 12374440

[B56] Mace M. L. (1950). *The Growth and Development of Executives.* Cambridge, MA: Harvard University.

[B57] Matsuo M. (2018). How does managerial coaching affect individual learning? The mediating roles of team and individual reflexivity. *Pers. Rev.* 47 118–132. 10.1108/pr-06-2016-0132

[B58] Maurer T. J. Pierce H. R. Shore L. M. (2002). Perceived beneficiary of employee development activity: a three-dimensional social exchange model. *Acad. Manag. Rev.* 27 432–444. 10.2307/4134388

[B59] McDaniel M. A. Schlager M. S. (1990). Discovery learning and transfer of problem-solving skills. *Cogn. Instr.* 7 129–159. 10.1207/s1532690xci0702_3

[B60] Mehmood Q. Hamstra M. R. W. (2016). Authentic leadership & follower performance outcomes: mediating role of learning goal orientation. *Acad. Manag. Annu. Meet. Proc.* 2016:10021.

[B61] Naveh E. Katz-Navon T. Stern Z. (2015). Active learning climate and employee errors: the moderating effects of personality traits. *J. Organ. Behav.* 36 441–459. 10.1002/job.2000

[B62] Noe R. A. Tews M. J. Dachner M. C. (2010). Learner engagement: a new perspective for enhancing our understanding of learner motivation and workplace learning. *Acad. Manag. Ann.* 4 279–315. 10.5465/19416520.2010.493286

[B63] Pan W. Sun L. Y. Chow I. H. S. (2011). The impact of supervisory mentoring on personal learning and career outcomes: the dual moderating effect of self-efficacy. *J. Vocat. Behav.* 78 264–273. 10.1016/j.jvb.2010.05.001

[B64] Parsloe E. Leedham M. (2009). *Coaching and Mentoring: Practical Methods to Improve Learning*, 2nd Edn London: Kogan Page.

[B65] Pham M. Avnet T. (2004). Ideals and oughts and the reliance on affect versus substance in persuasion. *J. Consum. Res.* 30 503–518. 10.1086/380285

[B66] Podsakoff P. M. MacKenzie S. B. Lee J.-Y. Podsakoff N. P. (2003). Common method biases in behavioral research: a critical review of the literature and recommended remedies. *J. Appl. Psychol.* 88 879–903. 10.1037/0021-9010.88.5.879 14516251

[B67] Redmond M. R. Mumford M. D. Teach R. (1993). Putting creativity to work: effects of leader behavior on subordinate creativity. *Organ. Behav. Hum. Decis. Process.* 55 120–151. 10.1006/obhd.1993.1027

[B68] Redshaw B. (2000). Do we really understand coaching? How can we make it work better? *Ind. Commer. Train.* 32 106–109. 10.1108/00197850010371693

[B69] Righetti F. Finkenauer C. Rusbult C. (2011). The benefits of interpersonal regulatory fit for individual goal pursuit. *J. Pers. Soc. Psychol.* 101 720–736. 10.1037/a0023592 21534700

[B70] Runhaar P. Bouwmans M. Vermeulen M. (2019). Exploring teachers’ career self-management. considering the roles of organizational career management, occupational self-efficacy, and learning goal orientation. *Hum. Resour. Manag.* 22 364–384. 10.1080/13678868.2019.1607675

[B71] Salancik G. R. Pfeffer J. (1978). A social information processing approach to job attitudes and task design. *Adm. Sci. Q.* 23 224–253. 10.2307/2392563 10307892

[B72] Shah J. Higgins E. T. Friedman R. S. (1998). Performance incentives and means: how regulatory focus influences goal attainment. *J. Pers. Soc. Psychol.* 74 285–293. 10.1037/0022-3514.74.2.285 9491583

[B73] Shariq S. M. Mukhtar U. Anwar S. (2018). Mediating and moderating impact of goal orientation and emotional intelligence on the relationship of knowledge oriented leadership and knowledge sharing. *J. Knowl. Manag.* 23 332–350. 10.1108/jkm-01-2018-0033

[B74] Sharma P. N. Kirkman B. L. (2015). Leveraging leaders: a literature review and future lines of inquiry for empowering leadership research. *Group Organ. Manag.* 40 193–237. 10.1177/1059601115574906

[B75] Shojaei S. H. Zakeri-Nia S. Najarzadeh-Arani S. BabaeiNejad R. F. (2016). The effect of transformational leadership on employee empowerment with the mediation of learning-oriented (a case study of Semnan University). *J. Adm. Manag. Educ. Train.* 12 48–61. 10.32861/jssr.51.48.50

[B76] Steelman L. A. Wolfeld L. (2018). The manager as coach: the role of feedback orientation. *J. Bus. Psychol.* 33 41–53. 10.1007/s10869-016-9473-6

[B77] Sue-Chan C. Wood R. E. Latham G. P. (2012). Effect of a coach’s regulatory focus and an individual’s implicit person theory on individual performance. *J. Manag.* 38 809–835. 10.1177/0149206310375465

[B78] Tang G. Kwan H. K. Zhang D. Zhu Z. (2016). Work–family effects of servant leadership: the roles of emotional exhaustion and personal learning. *J. Bus. Ethics* 137 285–297. 10.1007/s10551-015-2559-7

[B79] Taris T. W. Kompier M. A. J. De Lange A. H. Schaufeli W. B. Schreurs P. J. G. (2003). Learning new behavior patterns: a longitudinal test of Karasek’s active learning hypothesis among Dutch teachers. *Work Stress* 17 1–20. 10.1080/0267837031000108149

[B80] Theeboom T. Beersma B. van Vianen A. E. (2014). Does coaching work? A meta-analysis on the effects of coaching on individual level outcomes in an organizational context. *J. Posit. Psychol.* 9 1–18. 10.1080/17439760.2013.837499

[B81] Wood R. Kakebeeke B. Debowski S. Frese M. (2000). The impact of enactive exploration on intrinsic motivation, strategy, and performance in electronic search. *Appl. Psychol.* 49 263–283. 10.1111/1464-0597.00014

[B82] Yang H. Bednall T. C. Rijn M. V. (2016). Influence of HRM on knowledge sharing: the mediating role of learning goal orientation. *Acad. Manag. Annu. Meet. Proc.* 2016:12624 10.5465/ambpp.2016.12624abstract

